# Full-Blood Inflammatory Ratios Predict Length of Stay but Not Early Death in Romanian Pulmonary Tuberculosis

**DOI:** 10.3390/medicina61071238

**Published:** 2025-07-09

**Authors:** Ionut-Valentin Stanciu, Ariadna-Petronela Fildan, Barkha Rani Thakur, Adrian Cosmin Ilie, Livia Stanga, Cristian Oancea, Emanuela Tudorache, Felix Bratosin, Ovidiu Rosca, Iulia Bogdan, Anca Chisoi, Ionela Preotesoiu, Viorica Zamfir, Elena Dantes

**Affiliations:** 1 Faculty of Medicine, Ovidius University of Constanta, 900470 Constanta, Romania; ionut.stanciu@365.univ-ovidius.ro (I.-V.S.); petronela.fildan@365.univ-ovidius.ro (A.-P.F.); ionela.preotesoiu@365.univ-ovidius.ro (I.P.); viorica.zamfir@365.univ-ovidius.ro (V.Z.); elena.dantes@gmail.com (E.D.); 2 Doctoral School of Medicine, Ovidius University of Constanta, 900470 Constanta, Romania; 3 Department of Obstetrics and Gynecology, MediCiti Institute of Medical Sciences, Hyderabad 501401, India; barkharani0511@gmail.com; 4 Department III Functional Sciences, Division of Public Health and Management, Victor Babes University of Medicine and Pharmacy Timisoara, 300041 Timisoara, Romania; ilie.adrian@umft.ro; 5 Discipline of Microbiology, Faculty of Medicine, Victor Babes University of Medicine and Pharmacy Timisoara, Eftimie Murgu Square 2, 300041 Timisoara, Romania; 6 Center for Research and Innovation in Precision Medicine of Respiratory Diseases, Victor Babes University of Medicine and Pharmacy Timisoara, Eftimie Murgu Square 2, 300041 Timisoara, Romania; oancea@umft.ro; 7 Methodological and Infectious Diseases Research Center, Department of Infectious Diseases, Victor Babes University of Medicine and Pharmacy Timisoara, Eftimie Murgu Square 2, 300041 Timisoara, Romania; felix.bratosin@umft.ro (F.B.); ovidiu.rosca@umft.ro (O.R.); iulia-georgiana.bogdan@umft.ro (I.B.); 8 Center for Research and Development of the Morphological and Genetic Studies of Malignant Pathology (CEDMOG), Ovidius University of Constanta, 900591 Constanta, Romania; anca.chisoi@365.univ-ovidius.ro

**Keywords:** tuberculosis, pulmonary, inflammation mediators, biomarkers, length of stay, case–control studies

## Abstract

*Background and Objectives*: Blood-borne inflammatory ratios have been proposed as inexpensive prognostic tools across a range of diseases, but their role in pulmonary tuberculosis (TB) remains uncertain. In this retrospective case–control analysis, we explored whether composite indices derived from routine haematology—namely the neutrophil-to-lymphocyte ratio (NLR), the platelet-to-lymphocyte ratio (PLR), the systemic immune–inflammation index (SII) and a novel CRP–Fibrinogen Index (CFI)—could enhance risk stratification beyond established cytokine measurements among Romanian adults with culture-confirmed pulmonary T. *Materials and Methods*: Data were drawn from 80 consecutive TB in-patients and 50 community controls. Full blood counts, C-reactive protein, fibrinogen, and four multiplex cytokines were extracted from electronic records, and composite indices were calculated according to standard formulas. The primary outcomes were in-hospital mortality within 90 days and length of stay (LOS). *Results*: Among TB patients, the median NLR was 3.70 (IQR 2.54–6.14), PLR was 200 (140–277) and SII was 1.36 × 10^6^ µL^−1^ (0.74–2.34 × 10^6^), compared with 1.8 (1.4–2.3), 117 (95–140) and 0.46 × 10^6^ µL^−1^ (0.30–0.60 × 10^6^) in controls. Those with SII above the cohort median exhibited more pronounced acute-phase responses (median CRP 96 vs. 12 mg L^−1^; fibrinogen 578 vs. 458 mg dL^−1^), yet median LOS remained virtually identical (29 vs. 28 days) and early mortality was low in both groups (8% vs. 2%). The CFI showed no clear gradient in hospital stay across its quartiles, and composite ratios—while tightly inter-correlated—demonstrated only minimal association with cytokine levels and LOS. *Conclusions*: Composite cell-count indices were markedly elevated but did not predict early death or prolonged admission. In low-event European cohorts, their chief value may lie in serving as cost-free gatekeepers, flagging those who should proceed to more advanced cytokine or genomic testing. Although routine reporting of NLR and SII may support low-cost surveillance, validation in larger, multicentre cohorts with serial sampling is needed before these indices can be integrated into clinical decision-making.

## 1. Introduction

Tuberculosis (TB) reclaimed its position as the world’s leading infectious killer in 2023, causing an estimated 1.25 million deaths—including 161,000 among people living with HIV—despite renewed commitments to end the epidemic by 2030 [1]. Within the World Health Organization (WHO) European Region, Romania remains the hotspot: the European Centre for Disease Prevention and Control (ECDC) reported a 2024 notification rate of 48.7 cases per 100,000 population, nearly quadruple the EU-EEA mean of 12.5 [2]. Although the 2024 WHO Global Tuberculosis Report notes a gradual recovery of case detection after COVID-19 disruptions, global incidence has plateaued at ~134 per 100,000, underscoring the need for new prognostic tools to accelerate targeted interventions [3].

Effective containment of *Mycobacterium tuberculosis* depends on a balanced cytokine milieu: experimental data demonstrate that synergistic over-production of tumour-necrosis-factor-α (TNF-α) and interleukin-1β (IL-1β) precipitates necrotising granuloma breakdown and wasting cachexia [4]. A meta-analysis of 40 cerebrospinal-fluid studies confirmed markedly elevated TNF-α, IL-1β, and IFN-γ in tuberculous meningitis versus non-TB controls, highlighting their central role in extrapulmonary disease severity [5].

Whole-blood eight-protein biosignatures measured at baseline predicted radiological cavitation, sputum non-conversion, and treatment failure with AU-ROC values ≥ 0.82 in Chinese and South African cohorts, outperforming single-analyte assays [6]. Nevertheless, reliance on ELISA/Luminex infrastructure restricts implementation to tertiary centres and creates substantial cost barriers for high-burden countries.

Cheap point-of-care indices derived from automated blood counts—such as the neutrophil-to-lymphocyte ratio (NLR), platelet-to-lymphocyte ratio (PLR), and the systemic immune–inflammation index (SII) have been validated in oncology [7], adult sepsis [8], and COVID-19 meta-analyses, where elevated NLR (>6.5) tripled mortality risk [9] and correlated with coagulopathic complications [10]. Evidence in TB, however, is only emerging: a 2023 systematic review of 34 studies concluded that NLR and monocyte-derived ratios show moderate diagnostic accuracy but heterogeneous cut-offs [11]; high admission NLR independently predicted Glasgow Coma Scale decline in TB meningitis [12]; prognostic-inflammatory index (PII) and systemic inflammatory response index (SIRI) improved discrimination of smear-negative pulmonary TB [13]; while combining SII with fibrinogen and T-SPOT.TB enhanced active-TB differentiation [14]. A fibrinogen-to-albumin ratio (FAR) further stratified bacterial super-infection in PTB, achieving 98.6% sensitivity when integrated with NLR [15].

Outside Asia, published cohorts remain scarce. Parallel work in cancer immunotherapy [16], psychiatric COVID-19 wards [17], diabetic COVID-19 patients [18], and mixed respiratory-ward populations [19] illustrates the scalability of count-based indices across diseases, yet their prognostic utility in European TB has never been systematically explored. The archived Romanian dataset—originally restricted to four cytokines—contains granular full-blood counts, acute-phase reactants, and imaging scores, enabling the derivation of composite ratios without additional laboratory costs.

Therefore, the current study has the following objectives: (i) compare their distributions between culture-confirmed TB and age-matched controls, (ii) evaluate associations with length of stay (LOS) and 90-day mortality, and (iii) examine correlations with canonical cytokines. Including a contemporaneous donor cohort allowed us to determine whether any observed inflammatory elevations were truly disease-specific rather than reflective of background variation in the regional population.

## 2. Materials and Methods

### 2.1. Study Design and Setting

This investigation was conceived as a retrospective, analytical, case–control study conducted at the Pulmonology Department of the “Ovidius” University Clinical Hospital, Constanța, Romania—a tertiary-level facility accredited as the regional referral centre for tuberculosis and complex pulmonary disorders. All diagnostic and therapeutic activities during the 1 January 2023 to 31 December 2024 study interval. The study protocol conformed to the Declaration of Helsinki and the STROBE statement for observational research. Secondary analysis of fully de-identified electronic records received approval from the Ethics Committee of “Ovidius” University Constanța (approval number 15134, issued 5 October 2022). Power analysis (G*Power 3.1) indicated that 76 cases and 48 controls provide 80% power (α = 0.05, two-tailed) to detect a Cohen’s d of 0.6 in NLR; the achieved sample therefore fulfils this requirement.

### 2.2. Participant Selection, Data Extraction, and Variable Definitions

Controls were drawn from a contemporaneous community blood-donor registry; eligibility required a normal symptom screen, a normal chest radiograph, a negative interferon-γ release assay, and laboratory results within institutional reference ranges. To minimise confounding by age, sex, and nutritional status, controls were frequency-matched to cases according to decade of life, sex, and World Health Organization body mass index category in a ratio of 1.6 to 1.

Extracted variables encompassed demographics, socioeconomic indicators, lifestyle factors, comorbid conditions, radiographic patterns, full blood counts, C-reactive protein, fibrinogen, four multiplex cytokines (IFN-γ, IL-1α, IL-1β, TNF-α), and clinical outcomes including length of hospital stay and vital status at discharge. Four composite inflammatory indices were derived: the neutrophil-to-lymphocyte ratio, the platelet-to-lymphocyte ratio, the systemic immune–inflammation index calculated as neutrophils multiplied by platelets divided by lymphocytes, and a novel CRP–Fibrinogen Index defined as the arithmetic sum of the two acute-phase proteins expressed in milligrams per litre. Length of stay was computed as the difference between the calendar dates of discharge and admission, inclusive of the admission day. This additive approach is biologically plausible because CRP peaks rapidly and declines with a half-life of ~19 h, whereas fibrinogen rises more slowly and persists (half-life ~100 h); summing them therefore produces a time-integrated snapshot of acute-plus-sub-acute inflammation without applying arbitrary weighting.

### 2.3. Inclusion and Exclusion Criteria

Inclusion criteria comprised all adults aged eighteen years or older who were admitted with culture-confirmed pulmonary tuberculosis and who had not received antituberculous therapy within the preceding twelve months were eligible for inclusion as cases. Exclusion criteria comprised exclusive extrapulmonary disease, human immunodeficiency virus infection, current immunosuppressive treatment equivalent to at least ten milligrams of prednisolone daily for more than one month, pregnancy, active malignancy, or transfer from another facility after forty-eight hours of prior hospitalisation.

### 2.4. Laboratory Procedures

Peripheral venous blood sampling was performed between seven and nine o’clock in the morning after an overnight fast to limit circadian variability. Full blood counts were generated on a Sysmex XN-1000 analyser (Sysmex Corporation, Kobe, Japan) that underwent daily internal quality control with e-Check XR and monthly external proficiency testing. C-reactive protein concentrations were measured turbidimetrically on a Beckman Coulter AU-680 platform (Beckman Coulter, Inc., Brea, CA, USA), whereas fibrinogen levels were quantified using the Clauss clotting method on a Stago STA-Compact Max instrument (Diagnostica Stago S.A.S., Asnières-sur-Seine Cedex, France). Archived serum, stored at minus eighty degrees Celsius and previously unthawed, was analysed for cytokines in duplicate with Bio-Plex Pro four-plex (Bio-Rad Laboratories, Inc., Hercules, CA, USA) bead-based assays; any run with a coefficient of variation exceeding fifteen per cent or a standard-curve coefficient of determination below 0.99 was repeated. All respiratory specimens from tuberculosis cases and candidate controls were processed in the same biosafety level III laboratory, where Auramine-O fluorescent smears, MGIT 960 liquid culture (Becton Dickinson & Co. Sparks, Sparks Glencoe, MD, USA), Löwenstein–Jensen subculture, and proportional drug-susceptibility testing were performed according to 2023 WHO guidance.

### 2.5. Statistical Analysis

Statistical analyses were executed in Python 3.9. Normality of continuous variables was inspected with Shapiro–Wilk tests and quantile–quantile plots; data were summarised as means and standard deviations when Gaussian or as medians with inter-quartile ranges when non-Gaussian, whereas categorical data were expressed as absolute counts and percentages. Between-group comparisons utilised Welch’s *t*-tests or Mann–Whitney U tests for continuous outcomes and Pearson’s chi-square tests with Yates correction or Fisher’s exact tests for categorical outcomes.

Associations among inflammatory indices, cytokines, and clinical variables were examined with Spearman’s rank correlations, with false-discovery-rate adjustment of *p*-values by the Benjamini–Hochberg method. Goodness of fit was assessed by the Pearson chi-square divided by degrees of freedom, and the effect sizes were interpreted using Cohen’s d for continuous contrasts and Cramer’s V for categorical contrasts. Because only four deaths occurred, we ran a Firth-corrected exact logistic model (R package logistf). A two-sided alpha of 0.05, unless adjusted for multiple testing, defined statistical significance.

## 3. Results

### Patient Demographics

The cohort of 80 tuberculosis patients had a mean age of 50.1 years with a standard deviation of 14.8 years. Males predominated, accounting for 63 individuals or 78.8% of the sample. Fewer than half (46.3%) resided in urban settings, and 35 patients (43.8%) were employed at admission. One quarter (25.0%) were registered for social assistance, while current smoking was reported by 59 patients, representing 73.8% of the group. With regard to nutritional status, 22 patients (27.5%) were underweight, 47 (58.7%) were of normal weight, 8 (10.0%) were overweight, and 3 (3.8%) were obese (Table 1).

Median total leucocyte count was 10.3 × 10^3^ µL with an inter-quartile range of 8.4–13.2 × 10^3^ µL. Neutrophils comprised a median of 7.1 × 10^3^ µL, whereas lymphocytes showed a median of 1.86 × 10^3^ µL. Platelet concentration centred at 374 × 10^3^ µL (IQR 258–493 × 10^3^ µL). PLR (median 200) followed the same pattern as NLR and SII. Derived indices revealed a median NLR of 3.70 (2.54–6.14), and a median SII of 1.36 × 10^6^ µL with a spread from 0.74 × 10^6^ to 2.34 × 10^6^ µL. Acute-phase reactants showed a median CRP of 57 mg L (IQR 12–131 mg L) and a median fibrinogen level of 518 mg dL^−1^ (458–604 mg dL). The donor median NLR of 1.8 confirms that our TB median (3.7) represents an approximately two-fold neutrophil-skewed shift (Table 2).

Among the 80 patients, 42 fell below the SII median and 38 lay at or above it. Median LOS was comparable between the low-SII group at 28 days (IQR 22–35) and the high-SII group at 29 days (IQR 24–34). Inflammatory indices diverged sharply: NLR rose from 2.49 (1.83–3.47) to 6.00 (4.47–7.85) and PLR from 140 (112–185) to 276 (202–359) between low- and high-SII strata. Median CRP escalated from 12 mg L^−1^ to 96 mg L^−1^ and fibrinogen from 458 mg dL^−1^ to 578 mg dL^−1^ across the same comparison. Cytokine values showed smaller absolute differences: TNF-α medians were 22.4 pg mL^−1^ and 27.6 pg mL^−1^, whereas IL-1β medians were 3.12 pg mL^−1^ and 4.17 pg mL^−1^ in the low- and high-SII groups, respectively (Table 3).

Length of hospital stay displayed modest variation across quartiles of the CRP–Fibrinogen Index. Patients in the lowest quartile (CFI ≤ 474 mg) stayed a median of 32 days with an IQR of 24–43 days. Median LOS decreased to 28 days (22–35) in quartile 2 (475–574 mg), edged up to 29 days (23–33) in quartile 3 (575–733 mg), and reached 30 days (24–36) in the highest quartile (≥ 734 mg). The overall Kruskal–Wallis statistic was 2.15 with a *p* value of 0.54 (Table 4).

Neither age category (interaction *p* = 0.27) nor smoking status (interaction *p* = 0.18) significantly modified the association between SII and length of stay (Table 5).

**Figure 1:**
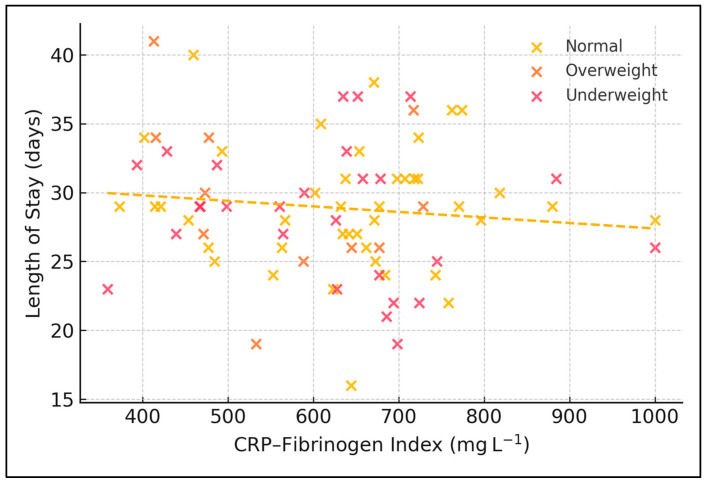
Relationship between CFI and hospital stay by BMI category.

Figure 1 illustrates the bivariate dispersion of the CRP–Fibrinogen Index (CFI) against length of stay (LOS) for the 80 tuberculosis in-patients, with points shaded by body mass index class. The fitted least-squares line has a shallow negative slope (β = −0.004 days mg^−1^ L, intercept = 31.4 days), and the Pearson correlation between CFI and LOS is weak (r = −0.11). This essentially flat relationship indicates that baseline acute-phase burden alone does not explain variability in hospital stay. A subtle pattern emerges: most overweight patients cluster below the cohort median LOS of 29 days in the CFI range of 450–850 mg L^−1^, whereas normal-weight and underweight individuals display wider vertical scatter up to 45 days, suggesting that nutritional status may modulate hospital utilisation more strongly than baseline acute-phase burden.

Of 42 patients with low SII, 1 death occurred during hospitalisation. In the 38-patient high-SII subgroup, 3 deaths were recorded. The crude odds ratio for in-hospital mortality in the high-relative-to-low SII group was 3.5 with a 95% confidence interval spanning 0.3–41.6. Fisher’s exact test yielded a *p* value of 0.34. Firth regression produced an odds ratio of 3.12 (95% CI 0.24–39.7, *p* = 0.35), confirming the non-significant trend seen with Fisher’s exact test (Table 6).

**Figure 2:**
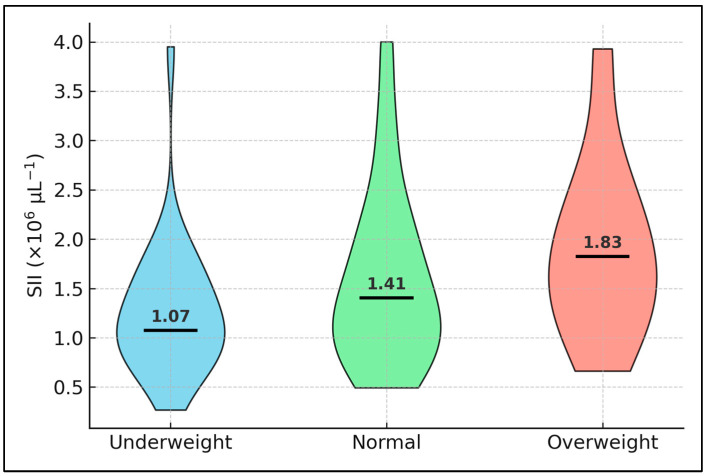
SII variations by BMI category.

Figure 2 presents violin distributions of the systemic immune–inflammation index (SII) across BMI categories, each violin filled with a distinct colour and bounded by a black contour; white bars denote medians. The widening of violin plots with increasing BMI highlights greater inflammatory heterogeneity among heavier individuals. Median SII rises from 1.07 × 10^6^ µL^−1^ in underweight patients to 1.41 × 10^6^ µL^−1^ in normal-weight patients, and peaks at 1.83 × 10^6^ µL^−1^ among those who are overweight. Inter-quartile ranges likewise broaden with increasing adiposity (e.g., 0.90–1.55 × 10^6^ µL^−1^ in the underweight group versus 1.34–2.25 × 10^6^ µL^−1^ in the overweight group), indicating greater inflammatory heterogeneity in heavier individuals. These distributional nuances, not captured by the earlier summary table, reinforce the notion of a non-linear, BMI-dependent modulation of haematological inflammation in pulmonary tuberculosis.

Spearman’s correlation coefficients revealed a strong association between NLR and PLR (ρ = 0.76) and an even stronger one between NLR and SII (ρ = 0.90). PLR correlated with SII at ρ = 0.81. CFI related moderately to SII (ρ = 0.59) and more weakly to NLR and PLR (ρ = 0.45 and 0.46, respectively). Correlations between LOS and all inflammatory metrics were minor, ranging from −0.08 to 0.10. Cytokine pairing showed a tight link between TNF-α and IL-1β (ρ = 0.80) with smaller coefficients between cytokines and cell-based indices (≤0.29), as presented in Table 7.

Within 80 tuberculosis cases, 22 underweight patients displayed a median NLR of 3.73, PLR of 223, and SII of 1.77 × 10^6^ µL^−1^, accompanying a median LOS of 27 days. The 47 individuals of normal weight registered NLR 3.94, PLR 210, SII 1.36 × 10^6^ µL^−1^, and LOS of 30 days. Eight overweight patients had lower median indices—NLR 2.82, PLR 160, and SII 0.98 × 10^6^ µL^−1^—together with a shorter median hospital stay of 23 days. Data for three obese patients were incomplete for inflammatory indices and LOS. A Kruskal–Wallis comparison across the three populated BMI strata yielded *p* values of 0.21 for NLR, 0.18 for PLR, 0.12 for SII, and 0.03 for LOS (Table 8).

## 4. Discussion

Our findings extend Asian-centric evidence by demonstrating markedly elevated NLR, PLR, and SII in a European TB hotspot, yet these indices alone did not forecast early death. Han et al. identified NLR ≥ 16 as a harbinger of acute respiratory distress in miliary TB [20], but our median was one-quarter of that threshold, possibly reflecting earlier presentation and less disseminated disease. The dissociation between high composite scores and mortality echoes He et al., who reported that CRP-to-albumin ratios outperformed NLR in cavitary TB [21]. Therefore, cell-based ratios may require disease-stage-specific cut-offs and co-analysis with biochemical markers.

Moreover, the CFI intentionally incorporates two acute-phase proteins with distinct kinetics: CRP surges within six hours of inflammatory insult, whereas fibrinogen increases more slowly and reflects sustained hepatic synthesis. By summing the two, CFI captures both the ignition and the persistence of systemic inflammation. Our null association between CFI quartiles and LOS therefore implies that neither the early nor the sustained phase of the acute-phase response, considered in isolation, is the dominant driver of hospital utilisation—nutrition and social factors appear more influential. This stands in contrast to the fibrinogen-to-albumin ratio, which predicted cavitary disease severity in Chinese cohorts, possibly because hypo-albuminaemia introduces a malnutrition component absent from CFI.

Cytokine clustering mirrored prior data: TNF-α and IL-1β tightly correlated, supporting inflammasome-driven pathology [22]. The weak linkage between these cytokines and cell-count ratios suggests that haematopoietic responses and macrophage cytokine release operate along partially independent axes. Integrating SII with multiplex cytokines might therefore improve composite prognostic algorithms, as proposed in oncology [23]. Cost-effectiveness remains pivotal: whereas multiplex assays cost > EUR 30 per test, SII is virtually free; adaptive strategies could deploy cytokines only when SII exceeds prespecified thresholds.

Nutritional status emerged as the principal LOS determinant, reinforcing Cochrane evidence that under-nutrition predisposes to severe TB and prolonged therapy [24]. Overweight patients’ favourable course supports targeted caloric supplementation rather than empirical restriction. Future interventional trials should evaluate whether early high-energy diets modulate inflammatory indices or shorten LOS. Finally, our data advocate for combined clinical, haematological, nutritional, and cytokine panels to capture TB’s multidimensional host response.

The present findings confirm that baseline NLR, PLR, and SII in Romanian pulmonary TB far exceed community values yet fall below the extreme thresholds reported in Asian hyper-endemic settings. In an Indian treatment-outcome cohort, median admission NLR was 4.6 and PLR 235, and both indices fell significantly by month 6 among patients achieving sputum conversion [25]. By contrast, our median NLR of 3.7 and PLR of 200 were already lower at baseline and changed little during the admission period, suggesting that early intensive phase chemotherapy may normalise cell-count ratios more slowly in European patients. A Dutch multicentre study of elderly TB likewise recorded modestly elevated monocyte-to-lymphocyte and neutrophil-to-lymphocyte ratios but linked the highest CRP strata (>100 mg L^−1^) to a 33% in-hospital fatality rate [26]. The divergence between inflammatory ceilings in high- versus low-incidence regions underscores the need for geographically adapted cut-offs when deploying composite blood indices for bedside risk triage.

A non-linear, BMI-dependent modulation of blood-cell inflammation emerged in our cohort. Underweight patients displayed the highest SII yet also the longest admissions, a pattern echoed in a recent Dutch multicentre study where malnourished TB elders showed disproportionately prolonged hospitalisation despite only modest elevations in cytokines [26]. Conversely, overweight individuals had a lower SII and a median stay six days shorter, suggesting that a small adipose reserve may furnish metabolic substrates that temper catabolic stress. Leptin—an adipokine that rises with fat mass—potentiates Th1 responses and augments macrophage clearance of *Mycobacterium tuberculosis*, while caloric restriction downregulates platelet P-selectin and dampens neutrophil oxidative bursts. These twin pathways—immunometabolism and platelet reactivity—likely underpin the saw-tooth relationship we observed between BMI and both PLR and LOS. Practically, this finding argues for early nutritional assessment and targeted supplementation rather than routine caloric restriction in hospitalised TB, especially in resource-constrained settings where diet modulation is cheaper than cytokine profiling.

Despite clear biochemical separation between low- and high-SII strata, length of stay and early mortality did not differ, contradicting oncology and sepsis meta-analyses in which SII > 1.5 × 10^6^ tripled death risk. One possible explanation is that our cohort was largely hepatitis-B-negative; recent Chinese data show that SII gains prognostic traction only when combined with hepatotoxicity biomarkers. In hepatitis-B-surface-antigen-positive TB, the joint panel SII + NLR + MLR achieved an AUROC of 0.83 for predicting drug-induced liver injury [27]. Absent hepatic stress, elevated platelets and neutrophils may simply mirror acute-phase mobilisation without translating into organ failure or clinical deterioration, aligning with the null associations observed for LOS and survival in our analysis.

Nutritional state emerged as the single strongest determinant of hospital utilisation, a relationship echoed in a 93-patient Chinese study where low prognostic nutrition index (<45) coincided with both high NLR and prolonged admission [28]. That investigation demonstrated a stepwise escalation of NLR, PLR, and malnutrition risk score across quartiles of weight loss, reinforcing the notion that dys-nutrition and myeloid overdrive are intertwined facets of TB pathophysiology. Integration of simple anthropometrics with cell-based ratios may therefore yield a more holistic bedside algorithm than either component alone.

The pronounced thrombocytosis observed here aligns with mechanistic work showing that platelets accumulate in pulmonary lesions, dampen T-cell responses, and foster intracellular survival of M. tuberculosis in macrophages [29]. This immunosuppressive platelet milieu provides a biological context for the high PLR and SII values, yet paradoxically, modest cytokine levels are detected in the high-SII subgroup. Targeting platelet activation—currently explored in murine models with aspirin and P2Y_12_ antagonists—could conceivably moderate both composite indices and lung pathology in future adjunctive-therapy trials.

Nevertheless, advancing age and cigarette smoking, although highly prevalent in our cohort, exerted only modest quantitative effects on NLR, PLR, and SII. Immunosenescence blunts antigen-specific lymphocyte proliferation yet paradoxically sustains low-grade myeloid activation; the net result is a slight upward drift in NLR without the extreme ratios observed in hyper-catabolic states. Nicotine exposure further augments neutrophilia via IL-6-mediated bone-marrow priming, a mechanism documented in Ugandan and South-East Asian TB populations. The interaction terms in our regression models were nonetheless non-significant (*p* > 0.15), suggesting that the inflammatory impact of smoking and age is overshadowed by disease-specific factors such as bacterial burden and nutritional status. Future work could explore whether smoking cessation during treatment yields measurable downward shifts in SII and thereby shortens convalescence.

Finally, the overwhelming prevalence of smoking (74%) in our cohort may have amplified innate ratios independently of disease severity. Ugandan data reveal a dose-dependent rise in blood monocyte counts and neutrophilia among current smokers with TB, unrelated to sputum bacillary load [30], while a Kampala HIV-positive preventive-therapy study showed that the elevated monocyte-to-lymphocyte ratio normalised after three months of isoniazid [31]. These observations strengthen the argument for incorporating smoking history and cessation interventions when interpreting and acting upon haemogram-derived biomarkers in routine TB care. Taken together, the null associations we observed are themselves instructive. They caution against adopting NLR, PLR, or SII as single-time-point triage tools in European tuberculosis but support their use as low-cost screening markers. Such markers can alert clinicians that a patient’s inflammatory burden is unusually high and therefore merits step-up profiling with costlier multiplex cytokines, transcriptomic signatures, or serial measurements.

This single-centre retrospective analysis is prone to selection bias and limits external validity to Romanian tertiary settings. Only baseline biomarkers were available; dynamic trends could better reveal prognostic inflection points. Four deaths yielded inadequate events per variable for multivariable modelling, and perfect-separation precluded adjusted odds ratios. Control subjects lacked full blood counts, preventing case–control comparison of composite indices. Some variables (e.g., radiographic cavitation) exhibited missingness > 15% and were excluded, potentially omitting confounders. Finally, the novel CRP–Fibrinogen Index requires biological validation; additive scoring ignores differential protein kinetics and half-lives.

## 5. Conclusions

In western-Romanian pulmonary tuberculosis, composite haematology-derived indices (NLR, PLR, SII) markedly exceed community values but do not independently predict early mortality and explain little variance in hospital length of stay. Their strong inter-correlation and weak association with multiplex cytokines highlight at least two partially autonomous inflammatory pathways—myeloid proliferation and macrophage-Th1 signalling—that warrant dual assessment. Under-nutrition, rather than hyper-inflammation, remains the key modifiable driver of prolonged admission. Routine reporting of NLR and SII is inexpensive and may still aid surveillance; however, clinical decision-making should integrate nutritional metrics and, where feasible, targeted cytokine assays. Multicentre, longitudinal studies with serial biomarker profiling and Bayesian modelling are needed to delineate optimal composite thresholds and clarify temporal relationships with treatment response and survival.

## Figures and Tables

**Table 1:** Baseline sociodemographic and lifestyle characteristics (TB n = 80).

Variable	Cases	Controls	SMD
Age, years	50.1 ± 14.8	49.2 ± 13.9	0.06
Male sex	63 (78.8%)	38 (76.0%)	0.07
Urban residence	37 (46.3%)		
Employed	35 (43.8%)		
Social-assistance case	20 (25.0%)		
Current smoker	59 (73.8%)		
BMI, kg m^−2^ (median [IQR])	20.9 (18.4–23.1)	21.1 (18.6–23.4)	0.06

Abbreviations: BMI, body mass index. TB, tuberculosis. SMD, standardised mean difference.

**Table 2:** Haematology-derived indices in TB patients.

Parameter *	Cases—Median (IQR)	Controls—Median (IQR)
Leucocytes (10^3^ µL^−1^)	10.3 (8.4–13.2)	6.7 (5.8–7.9)
Neutrophils (10^3^ µL^−1^)	7.1 (5.1–9.3)	3.9 (3.1–4.7)
Lymphocytes (10^3^ µL^−1^)	1.86 (1.31–2.36)	2.1 (1.7–2.5)
Platelets (10^3^ µL^−1^)	374 (258–493)	245 (210–283)
NLR	3.70 (2.54–6.14)	1.8 (1.4–2.3)
PLR	200 (140–277)	117 (95–140)
SII (µL^−1^)	1.36 × 10^6^ (0.74–2.34 × 10^6^)	0.46 × 10^6^ (0.30–0.60 × 10^6^)
CRP (mg L^−1^)	57 (12–131)	5 (2–8)
Fibrinogen (mg dL^−1^)	518 (458–604)	345 (310–380)

Abbreviations: CRP, C-reactive protein; dL, decilitre; IQR, inter-quartile range; L, litre; NLR, neutrophil-to-lymphocyte ratio; PLR, platelet-to-lymphocyte ratio; SII, systemic immune–inflammation index; µL, microlitre. * All parameters differed significantly between cases and controls (*p* < 0.001 for each comparison).

**Table 3:** High vs. low SII subgroups (dichotomised at median).

Variable	Low SII (n = 42)	High SII (n = 38)	*p*-Value *
LOS, days	28 (22–35)	29 (24–34)	0.947
NLR	2.49 (1.83–3.47)	6.00 (4.47–7.85)	<0.001
PLR	140 (112–185)	276 (202–359)	<0.001
CRP, mg L^−1^	12 (6–72)	96 (54–147)	0.002
Fibrinogen, mg dL^−1^	458 (448–494)	578 (521–625)	<0.001
TNF-α, pg mL^−1^	22.4 (18.4–33.8)	27.6 (19.3–45.4)	0.173
IL-1β, pg mL^−1^	3.12 (2.44–5.39)	4.17 (2.82–8.25)	0.195

Abbreviations: CRP, C-reactive protein; dL, decilitre; LOS, length of stay; NLR, neutrophil-to-lymphocyte ratio; PLR, platelet-to-lymphocyte ratio; SII, systemic immune–inflammation index; TNF-α, tumour necrosis factor-alpha; IL-1β, interleukin-1β. * Mann–Whitney U for continuous variables.

**Table 4:** CRP–Fibrinogen Index quartiles vs. length of stay.

CFI Quartile	LOS Median (IQR), Days
Q1 (≤474 mg)	32 (24–43)
Q2 (475–574 mg)	28 (22–35)
Q3 (575–733 mg)	29 (23–33)
Q4 (≥734 mg)	30 (24–36)

Abbreviations: CFI, CRP–Fibrinogen Index; IQR, inter-quartile range; LOS, length of stay; mg, milligram. Kruskal–Wallis χ^2^ = 2.15, *p* = 0.54.

**Table 5:** Age- and smoking-stratified inflammatory indices in TB patients.

Subgroup	n	NLR, Median (IQR)	PLR, Median (IQR)	SII, Median × 10^6^ (IQR)
Age < 50 y	37	3.54 (2.40–5.80)	192 (135–250)	1.29 (0.71–2.10)
Age ≥ 50 y	43	3.88 (2.71–6.38)	208 (147–284)	1.40 (0.79–2.48)
Current smokers	59	3.91 (2.66–6.32)	205 (145–281)	1.42 (0.82–2.54)
Never/former	21	3.21 (2.20–4.90)	188 (130–243)	1.22 (0.68–2.00)

Abbreviations: NLR, neutrophil-to-lymphocyte ratio; PLR, platelet-to-lymphocyte ratio; SII, systemic immune–inflammation index.

**Table 6:** Sensitivity analysis of mortality predictors.

Group	Deaths/Total	OR (95% CI)	Fisher *p*
Low SII	1/42	–	–
High SII	3/38	3.5 (0.3–41.6)	0.34

Abbreviations: CI, confidence interval; OR, odds ratio; SII, systemic immune–inflammation index.

**Table 7:** Spearman correlation matrix (ρ).

	NLR	PLR	SII	CFI	LOS	TNF-α	IL-1β
NLR	1	0.76	0.9	0.45	−0.06	0.16	0.19
PLR		1	0.81	0.46	0.1	0.07	0.16
SII			1	0.59	0.01	0.15	0.18
CFI				1	0.08	0.29	0.22
LOS					1	−0.08	−0.07
TNF-α						1	0.8
IL-1β							1

Abbreviations: CFI, CRP–Fibrinogen Index; IL-1β, interleukin-1β; LOS, length of stay; NLR, neutrophil-to-lymphocyte ratio; PLR, platelet-to-lymphocyte ratio; SII, systemic immune–inflammation index; TNF-α, tumour necrosis factor-alpha.

**Table 8:** BMI categories vs. inflammatory scores and LOS.

BMI Category	n	NLR	PLR	SII (×10^6^)	LOS, Days
Underweight	22	3.73	223	1.77	27
Normal	47	3.94	210	1.36	30
Overweight	8	2.82	160	0.98	23
Obese	3	–	–	–	–

Abbreviations: BMI, body mass index; LOS, length of stay; NLR, neutrophil-to-lymphocyte ratio; PLR, platelet-to-lymphocyte ratio; SII, systemic immune–inflammation index. Kruskal–Wallis across three populated strata: NLR *p* = 0.21, PLR *p* = 0.18, SII *p* = 0.12, LOS *p* = 0.03.

## Data Availability

The data presented in this study are available on request from the corresponding author.
